# The Need for Systematic Reviews of Reasons

**DOI:** 10.1111/j.1467-8519.2011.01858.x

**Published:** 2011-04-27

**Authors:** Neema Sofaer, Daniel Strech

**Keywords:** review literature as topic, systematic review, ethics, bioethics, policy making, delivery of health care, human experimentation

## Abstract

There are many ethical decisions in the practice of health research and care, and in the creation of policy and guidelines. We argue that those charged with making such decisions need a new genre of review. The new genre is an application of the systematic review, which was developed over decades to inform medical decision-makers about what the totality of studies that investigate links between smoking and cancer, for example, implies about whether smoking causes cancer. We argue that there is a need for similarly inclusive and rigorous reviews of reason-based bioethics, which uses reasoning to address ethical questions. After presenting a brief history of the systematic review, we reject the only existing model for writing a systematic review of reason-based bioethics, which holds that such a review should address an ethical question. We argue that such a systematic review may mislead decision-makers when a literature is incomplete, or when there are mutually incompatible but individually reasonable answers to the ethical question. Furthermore, such a review can be written without identifying all the reasons given when the ethical questions are discussed, their alleged implications for the ethical question, and the attitudes taken to the reasons. The reviews we propose address instead the empirical question of which reasons have been given when addressing a specified ethical question, and present such detailed information on the reasons. We argue that this information is likely to improve decision-making, both directly and indirectly, and also the academic literature. We explain the limitations of our alternative model for systematic reviews.

## INTRODUCTION

### 1. Reason-based versus empirical bioethics

Reason-based bioethics uses reasoning to address normative questions, for example, about whether participants in a drug trial are morally entitled to have access to the trial drug after the trial.^1^ The methods of reason-based bioethics exclude conducting empirical studies, although this sub-field of bioethics does use the results of empirical studies. (To avoid oversimplification, we should point out that the authors of reason-based bioethics use disparate methods and have different intellectual backgrounds^2^ and varying competence at reasoning.) Most bioethics is philosophical;^3^ for many authors, ‘bioethics’ refers to reason-based bioethics. Whereas this paper concerns only reason-based bioethics, we need to contrast it with the newer field of empirical bioethics, which conducts qualitative or quantitative studies designed to answer empirical questions relevant to reason-based bioethics or policy-making. A typical research question might be: What are the attitudes and opinions of participants in US clinical trials about whether or not, and why, they should be ensured access, after the trial, to the trial drug, health care and information?^4^

Publications in reason-based bioethics rarely articulate their relevance to clinical decision-makers or policy-makers. This has led us to wonder if there is a way to make reason-based bioethics accessible to such decision-makers, given that the field addresses so many of the questions they face.

### 2. The classical systematic review

A systematic review answers a specific empirical research question. The question has a set form: it must refer to the population, the intervention or exposure, the comparison and the outcome. One question might be: ‘[POPULATION] In humans aged 18 or over, [INTERVENTION] does smoking more than 12 cigarettes a day, [COMPARISON] versus not smoking, [OUTCOME] increase the lifetime incidence of lung cancer?’ The systematic review answers the question based on the entire literature and tells us how confidently we should accept the answer; alternatively, it concludes that the question is not yet settled: further research is needed.

To write a systematic review, one first conducts a search designed to identify all the relevant publications. This is called an ‘exhaustive search’. The term is misleading, as a search designed to be exhaustive may fail to be so. Publications are considered relevant if, and only if, they meet pre-decided and explicit conditions for inclusion. The search should be reproducible and the written review should describe it in sufficient detail to enable its reproduction. The result of this search should be the entire literature that addresses the research question.

Next, one extracts from each selected publication the conclusion that it draws (its answer to the research question) and other data. One assesses the degree to which we should believe the answer/conclusion; sometimes, and as recommended when writing a systematic review that will inform clinical guidelines, one grades the answer/conclusion to reflect this degree.^5^ A particular study might give us strong reason to believe that smoking slightly increases cancer incidence; another might give us weak reason to believe that smoking greatly increases cancer incidence. The systematic review then concludes whether smoking increases cancer incidence, based on all the relevant publications, taking into account the extent to which we should accept each individual publication's answers/conclusions.

The primary purpose of the systematic review is to improve decisions: to enable decisions that are maximally informed and minimally biased. The need for systematic reviews arises because, for most empirical questions, the relevant literature is extensive. Decision-makers, for example clinicians and policymakers, lack the time and skills to retrieve all the relevant literature, appraise it and synthesize it in order to identify the relevant literature's all-things-considered answer to the research question. Without a systematic review, decisions are likely to be based on a subset of publications, which may not be representative of the whole literature, and the risk arises that the reviewers will consciously or unconsciously ‘cherry-pick,’ that is, select publications best supporting their views. Systematic reviews undertake the substantial project of identifying, assessing and synthesizing the literature using techniques that minimize bias, and they present the synthesis in a format accessible to decision-makers.^6^ Unsurprisingly, systematic reviews are used increasingly to make clinical decisions, write clinical guidelines and set research agendas.

We will call this the ‘classical systematic review’ to distinguish it from later applications. Two features make it classical: the fact that the review answers an empirical question of the specific form we described, and the process used to answer the question.

### 3. Newer applications of the systematic review

Systematic evaluation and synthesis emerged in the late 1970s in social science to address questions, for example, about the relation between class size and pupil achievement, and later spread to medicine and various fields including qualitative health research.^7^ Recently, one of us argued that the systematic review should be transferred to empirical bioethics, and proposed a model.^8^ Furthermore, Laurence McCullough and colleagues have argued that reason-based bioethics needs systematic reviews.^9^ They outlined a model and illustrated it by conducting a systematic review of a seven-article literature. We will call this outline model the ‘McCullough Model’. To our knowledge, this is the only model for systematic reviews of reason-based bioethics.

There are challenges to each of these newer applications. One of us has shown that a systematic review of empirical bioethics needs to address a different form of research question from the classical systematic review, and to use a modified search strategy.^10^ Similarly, Laurence McCullough and colleagues have highlighted challenges to searching for reason-based bioethics literature.

The rationale given for each new application is analogous to that for the classical systematic review. For example, McCullough and colleagues express concern that doctors may lack the skills to retrieve all of the relevant reason-based literature and to assess its quality, and that consequently, if there are no systematic reviews of reason-based literature, doctors' ethically relevant decisions are likely to be biased.^11^

We will next identify key features of the McCullough Model. Then, we will argue that systematic reviews based on it may comprise an insufficiently informative, and misleading, brief for decision-makers; and they are also of limited use to reason-based bioethicists, and to empirical scientists conducting research relevant to policy.

### 4. Key features of the McCullough Model

#### 4a. Same type of research question as classical systematic review

The model prescribes the same form of research question as the classical review. Consider McCullough and colleagues' research question: ‘In patients with mental disorders … is use of concealed medications in food or drink, rather than prescribing medications in the usual way or forcibly administering them, ethically justifiable?’^12^ The question mentions the population (patients with mental disorders), intervention (use of medications concealed in food or drink), comparison (prescribing medications in the usual way or forcibly administering them), and outcome (whether or not the intervention is ethically justifiable). In short, the question remains focused on outcomes or, in other words again, on the conclusions drawn by different publications, their answers to the research question.

Though it passes unremarked by McCullough and colleagues, one change does occur in the research question in the shift from clinical epidemiology to reason-based bioethics: a change in the nature of the outcome, from a physical outcome, for example increased mortality, to an ethical outcome, for example ethical justifiability.^13^

#### 4b. No need to extract detailed information on reasons

The type of research question that the McCullough Model prescribes can be answered without extracting each occurrence of a reason from every included publication. (We contrast the *occurrence* of a reason in a publication with a *type* of reason, which may have different occurrences in different publications.) Nor does answering the question require extraction of information on whether these reason occurrences were used to argue for or against the ethical view in question, or whether a specific occurrence of a reason for the view was accepted or rejected.

#### 4c. Need to assess degree to which we believe each publication's conclusion

Because a McCullough Model systematic review seeks to draw the literature's all-things-considered ethical conclusion, it needs to assess the extent to which we should believe the conclusions of individual publications. As acknowledged by McCullough and colleagues, the key challenge is that the methods for assessing the quality of an empirical study do not apply to moral reasoning. Their model proposes that, for each included publication, a systematic review of reason-based literature should assign a score (0, or 1) to each of the following:

1. Whether a focused question is stated
2. Whether a literature search was performed and the extent to which it was clearly described
3. The ‘quality of the ethical analysis and argument’ in the publication
4. Whether or not a conclusion is given and its clarity, and
5. Whether there is a ‘clear statement of the clinical application of the ethical analysis and argument and their conclusion’.^14^

One then sums these five scores to obtain the publication's overall score for ‘adequacy of … ethical analysis and argument’.^15^ An earlier paper by the same author team explains the factors that influence the ‘quality of the article's analysis and argument’ (point 3, above); this quality is said to depend on the ‘validity’ and ‘soundness’ of all the publication's reasons for its overall conclusion.^16^

In sum: the McCullough Model for writing systematic reviews of reason-based bioethics is a model for writing systematic reviews that address an ethical question based on the answers to this question given in the literature. Furthermore, writing such reviews requires measuring the quality of reasoning.

## THE NEED FOR SYSTEMATIC REVIEWS OF REASONS

We agree with McCullough and colleagues that, if the research question is an ethical question, the key challenge is to measure the quality of reasoning, because we need to assess the degree to which we should believe each publication's ethical conclusion. However, as we next argue, a systematic review of reason-based bioethics needs to address a different type of research question – just as a systematic review of empirical bioethics needs to address a different type of research question from a classical systematic review. This objection and others to the McCullough Model will comprise the first part of our case for our thesis that there is a need for *systematic reviews of reasons*, as we call them. We will then outline our alternative model for writing systematic reviews of philosophical bioethics, and explain why it is less vulnerable to the same types of objection. Next, we will defend our claims that reason-based bioethics needs reviews that are both of reasons and systematic, and that the reviews we advocate deserve to be called ‘systematic’. We will also outline their limitations and identify other necessary components of a decision-maker's brief.

We will thereby argue that systematic reviews of reasons are good tools, better than both informal reviews^17^ of reasons and McCullough Model systematic reviews, for promoting the following: (1) minimally biased, maximally informed decisions in policy-making and governance, health care and medical research,^18^ (2) research that informs policy-making, and thus indirectly aids decision-making, and (3) progress in reason-based bioethics. Our arguments show also why we all – the public, bioethicists, social scientists, and decision-makers – need systematic reviews of reasons in reason-based bioethics.

### 1. Our objections to the McCullough Model

If a literature omits reasons or inadequately presents reasons that are potentially strong, its all-things-considered ethical conclusion will be uninteresting. Objections arise even if a literature is comprehensive and uniformly excellent. The all-things-considered conclusion may be only one of several conflicting but individually reasonable conclusions that could be drawn from that set of reasons. The same set of reasons might better support a different conclusion given a change in the context or weighting of the reasons. In any case, the reasons underlying the literature's all-things-considered conclusion must be scrutinized prior to making clinical decisions or policy. Thus, we hypothesize, McCullough Model systematic reviews of reason-based bioethics potentially mislead decision-makers, who may fail to appreciate the previous points.

McCullough and colleagues might reply that a systematic review must include disclosures about factors that limit the degree to which we should believe its all-things-considered conclusion. They indeed concede that the burden of proof created by their review's all-things-considered conclusion is ‘modest’ given the reviewed literature's limitations^19^ and, in their discussion, they reject a key reason given in the reviewed literature for the conclusion that the majority of the reviewed publications draw. Nevertheless, we fear that policymakers may ignore or discount the disclosures and, in any case, may choose to accept the all-things-considered conclusion for lack of an alternative conclusion. As we will argue below, a full set of published reasons for or against the view in question is more useful than a conclusion that is inadequate – whether due to limitations of the literature reviewed or of the systematic review's methodology.

We also object to the McCullough Model's measure of the quality of reasoning. First, we present a superficial objection, which is that whether a specific instance of reasoning is adequate depends partly on the type of publication in which it appears: (1) greater detail is required in a philosophy article that scrutinizes a specific argument than in a policy report that considers many arguments for a specific view. (2) A good philosophy article can consider just one argument, but a good policy article must consider a range of reasons or else justify why it does not. (3) A good philosophy article need not present evidence and discuss its quality – it may proceed, for example, on the basis of hypothetical scenarios – but a good policy article must do both. (4) For many bioethics questions, the discussion occurs in various fields and genres, such as philosophy articles and policy pieces.

This objection is an unfair objection to the specific systematic review presented by McCullough and colleagues because the only publications it covers are *articles* in reason-based bioethics. However, the McCullough Model is intended to apply to reason-based bioethics in general. The objection is nonetheless superficial because it is possible to develop criteria for the quality of different types of publication: a publication would then receive its score of 0, or 1 according to the relevant criteria.

The remaining objections are not superficial. The second objection is that independent coders are likely to score differently the quality of reasoning in the same publication. The McCullough Model intends one number (0, or 1) to score both validity and soundness. However, it is unclear, and is left unclear, what score to assign to a valid argument with false premises: how good should we consider such an argument? Similarly, it is unclear, and is left unclear, how to score the quality of reasoning in a publication when it presents some reasons competently but not others. This objection can be avoided to some extent by measuring quality other than by using a summary measure; we note that the Cochrane Handbook, which is the main handbook for writing classical systematic reviews, ‘… recommend[s] against the use of scales yielding a summary score’.^20^

Nonetheless, a third objection, about the measurement of quality, remains. It may take considerable research to assess whether or not an argument's premises are all true: the truth-value of factual premises may be as yet unknown, and there may be reasonable disagreement about moral premises.

We therefore conclude that the McCullough Model's measure of the quality of reasoning in a publication is an inadequate indicator of the extent to which we should believe the publication's all-things-considered ethical conclusion. In any case, even if one found a satisfactory measure of quality, and used it to replace the Model's measure of quality, our (in our opinion, decisive) objections to the fact that the research question is supposed to be an ethical question would remain.

### 2. Key features of our alternative model

This section outlines our alternative model, which is a model for writing systematic reviews of reasons.

#### 2a. Different type of research question from classical systematic review

Our alternative model holds that a systematic review of reason-based bioethics should address not an ethical question, but the empirical question of which reasons have been given when the ethical question was addressed. More precisely, the research question should ask, for example, which reasons have been given for or against the view that the relevant intervention or action is morally justifiable, impermissible, permissible or obligatory, or for the views that a specific intervention should, or need not, be made.

To illustrate: we recently completed what we believe is the first systematic review of reasons, as well as the first systematic review of a large, mature reason-based bioethics literature.^21^

It addresses the question: ‘which reasons have been given for the views that former participants in a drug trial should, or need not, be ensured post-trial access to the trial drug?’ A secondary research question is: ‘how have these reasons been used to argue that post-trial access should, or need not, be ensured?’ These are factual questions about reasons used in ethical discussions and in drawing ethical conclusions, as distinct from corresponding conclusion-focused, ethical question that the McCullough team would instead address: ‘is it obligatory to ensure that former participants are given post-trial access to the trial drug?’ The latter could be rewritten in the standard form prescribed by the classical and McCullough Model systematic reviews, in terms of population, intervention, comparison and outcome. Note that, despite the fact that the literature to be reviewed is reason-based, the proposed research questions are empirical/descriptive, not ethical/normative.

For the sake of simplicity, we will use the case of post-trial access throughout this paper. However, it is important to point out that, for every ethical question, for example, whether abortion is permissible or whether there is a right to euthanasia, there is a corresponding reason-focused question. Our thesis that reason-based bioethics needs systematic reviews of reasons is not limited to any particular sub-field of reason-based bioethics. Similarly, although we will not elaborate on this point, many arguments in our case for the need for systematic reviews of reasons in bioethics apply to reason-based fields other than reason-based bioethics, such as law and parts of economics. This paper should be taken to present a case for systematic reviews of reasons more generally.

#### 2b. Need to extract detailed information on reasons

The form of the research question requires the collection of detailed information about reasons. For example, to answer our review's research questions about reasons why post-trial access should, or need not be ensured, authors should identify each reason occurrence in every included publication, and apply major and minor codes to each occurrence that refer respectively to the occurrence's broad type of reason and narrow type of reason. One broad type might be reasons based on the obligation to avoid exploitation; narrow types would include the obligation to avoid exploiting participants and the obligation to avoid exploiting countries that host research. Another broad type might be reasons based on legal requirements regarding post-trial access.^22^ One difference between the McCullough Model and ours is that our model proposes the extraction of detailed data on each occurrence of reason in each publication, while the McCullough Model proposes merely to assign 0, or 1 to an entire publication on the basis of its overall reasoning.

The authors of a systematic review should record also whether the reason occurrence is used to argue for or against the view in question: occurrences of the same reason type may have occurrences with different alleged implications.^23^ They should note whether the occurrence is accepted or rejected (or neither), because a commonly-mentioned type of reason may be never endorsed and never rejected, or always rejected.

After removing reasons that are repeated in a source, the authors should present data on (1) the broad and narrow types of reason given in the literature when the ethical question is discussed, (2) the number of occurrences of each broad and narrow type, (3) whether occurrences of the same type are used to argue for or against the view in question, or sometimes for and sometimes against, and (4) whether different occurrences of the same type that have the same alleged implication are accepted and/or rejected. It is crucial that the systematic review should contain a comprehensible taxonomy and visual presentation of all the reasons and their uses, including the implications that they have been taken to have for the research question. There are various options for such a presentation. We discuss these and present one elsewhere.^24^ Often, additional qualitative results will be appropriate. For example, the authors might compare and contrast the variants of the same reason given in different publications, and identify major trends regarding the attitudes taken to commonly-mentioned reasons.

The authors should also collect and present data on the publications that together comprise the included literature, for example, on the type of publication (such as article, chapter in edited book), year of publication and field of publication (such as medicine, philosophy). They should also list the included publications, and a table of the positions taken by each publication (the reasons endorsed and any final conclusion reached). Further details of the methodology we advocate are given elsewhere.^25^

#### 2c. No need to assess the degree to which we should believe each publication's conclusion

The form of the research question also implies that one does not need to assess the quality of reasons. However, information on quality is essential, in particular, to clinical decision-makers and policymakers, so we return to the issue of quality assessment later.

### 3. Why systematic reviews of *reasons*?

One might object that if a literature omits relevant reasons when addressing a question or presents potentially strong reasons inadequately – as do some literatures, in particular, young literatures – a systematic review of reasons will be as uninteresting as one of conclusions. We will next explain, using illustrations from our particular systematic review, why our proposed systematic reviews of reasons are useful even when the literature reviewed is uninteresting, and why they are, in general, less vulnerable to the objections we have advanced against the systematic reviews advocated by McCullough and colleagues. We will also explain why there is a need for systematic reviews of reasons, even though there are already informal reviews of reasons and reports promulgated by official bodies, which also offer decision-makers an intermediate option between reading all the reasons themselves and a summary of select, weighted reasons.

Before we proceed, it is crucial to point out that a systematic review of reasons cannot be the only item in a decision-makers' brief. Such a review does not help decision-makers to select the strong reasons from the published ones or to weight the strong reasons. Depending on the scope of the literature reviewed, it may also fail adequately to represent the arguments and views advanced by industry, regulators, research participants and other stakeholders. The brief should also include, among other items, a distillation of the strong reasons and their implications, and guidance on weighting these reasons that indicates, as appropriate, the possibility of drawing from these reasons, severally and jointly, alternative, individually reasonable conclusions. Yet, as the arguments below imply, distillation should occur only after a systematic review such as this one has captured all the published reasons and how they have been used.^26^ The systematic review remains an essential ingredient of a decision-makers' brief for the reasons above and because of decision-makers' need to check the distillation and its legitimacy.

By way of introduction, we will need key results from our systematic review: this identified 36 broad and 235 narrow types of reasons, based on a wide variety of moral, legal and practical considerations. None of the 75 publications included in the review reported more than 22 broad and 59 narrow types, even though the publication that reported these was an excellent informal review.^27^ The mean informal review reported only 24 narrow types of reason, compared to our review's 235.^28^ Among the publications were major reports by prominent bodies such as the US's National Bioethics Advisory Commission^29^ and the UK's National Council on Bioethics,^30^ which both failed to mention major families of reasons identified by our systematic review, as well as frequently published versions of specific reasons. The most-mentioned reason was avoiding exploitation of participants, their countries or communities (96 mentions). For this and many other reasons, publications differed about the reason's interpretation, implications and/or persuasiveness. Publications differed also on the facts about the costs, feasibility and legality of PTA.

These results are from a single – indeed, the only – systematic review of reasons. We hypothesize generally that: (1) current informal reviews of reason-based bioethics and reports produced by official bodies do not identify all the published reasons. (2) For any question in reason-based bioethics, a systematic review of reasons will identify a broader variety of published reasons, alleged implications of reasons and attitudes taken to reasons than searches typically conducted by those writing in reason-based bioethics or by the authors of official reports.

If these hypotheses are true, what is their significance? Policymakers considering, for example, whether or not to require that trial participants are ensured access to the trial drug after the trial must identify all the relevant reasons why post-trial access should, or need not be ensured to participants.^31^ Bioethicists seeking to understand when and why post-trial access should be ensured must do the same. As mentioned above, a systematic review of reasons should include a digestible visual presentation of all the published reasons and their alleged implications for the research question. The differences between our systematic review on the one hand, and informal reviews and reports on the other hand, suggest that such a presentation is currently the best tool that decision-makers and bioethicists have to ensure they do not overlook possibly relevant reasons and their alleged implications. In particular, a systematic review of reasons brings to light reasons that have been infrequently published and only inadequately presented, such as – in the case of our systematic review – rights-based reasons. If the publishing world works well, these less prominently published reasons will be a distraction. However, the most prominently published ones may simply be the best publicized, perhaps due to conflicts of interest that induce authors to endorse weak reasons and to ignore or reject strong reasons. In the absence of evidence either way, a systematic review based on a well-constructed search is crucial, in that it gives even such reasons an equal voice prior to selection and appraisal of the relevant reasons.^32^

Some reasons given in published discussions of the research question may, in fact, not be strong reasons or perhaps not even relevant to the research question. Although decision-makers and bioethicists need the strong reasons, a systematic review of reasons is still crucial. First, it is a minimally based starting point for identifying the strong reasons. Second, imagination, knowledge of reasons mentioned in other literatures, and analytical skills are not always optimal and, in any case, are sometimes insufficient to identify all the relevant reasons. Thought is stimulated by discovering differences between publications regarding the formulation, implications or persuasiveness of reasons, and differences in how often they are mentioned. Because the readers of a systematic review of reasons do not have to reinvent the wheels or, at any rate, need to invent fewer wheels, we surmise that they are less likely to miss relevant reasons and likelier to formulate relevant reasons plausibly and to understand their implications.^33^

There are further ways in which a systematic review of reasons is directly relevant to decision-making. Our systematic finding that the most-endorsed reasons included ones used just for ensuring PTA and others just for the view that PTA need not be ensured, provides a salutary warning to decision-makers against considering only reasons on one side of the case. Other systematic reviews of reasons are likely to provide similar warnings. Also, a systematic review may also imply that a procedural solution will be needed to set guidelines.^34^ Our systematic review suggested that reason-based bioethicists may fail to reach agreement, in that it showed that the most-endorsed reasons included ones used just for ensuring PTA, and others used just for the view that PTA need not be ensured.

Possible users of systematic reviews of reasons such as ours include the members of a committee, within a pharmaceutical company or government agency, charged with developing guidelines on post-trial access to trial drugs. NS's essential preparation for drafting the guidelines on post-trial access to trial drugs in collaboration with the UK's National Research Ethics Service included writing a systematic review of reasons why such access should, or need not be ensured.^35^ Such a review would also be useful for designers of research protocols such as researchers, research sponsors, and Contract Research Organizations, particularly given the fact that current guidance on post-trial access is inconsistent, ambiguous and silent on many aspects of post-trial access.^36^ The public needs these other parties to read systematic reviews of reasons.

We surmise that a systematic review of reasons is also indirectly relevant to decision-making, in that it is a particularly good tool for inspiring and guiding empirical and conceptual research that improves the information base of decisions.^37^ Various examples follow. First, such a review may identify moral views that differ between publications or indeed trends in attitudes taken to a specific view. Our review identified a trend towards rejecting the view that avoiding exploitation requires post-trial access. This view was common in the early literature and assumed by prominent guidelines written during this early stage (see e.g.^38^). Thus, our review suggested the need to scrutinize these guidelines. Second, a systematic review may identify, as did ours, reasons that have been presented only inadequately. Third, as mentioned, identifying all the different published formulations of a reason will better enable the selection of the relevant one(s). Fourth, such a review can also show, as did ours, that many authors, who used specific interpretations of a reason, did not express awareness of some or any other interpretations, or relevant literature in the same and other fields, and that some of the reasoning is incomplete or appears invalid. Fifth, the stark differences that our systematic review identified in authors' use of terms for key concepts such as exploitation and reciprocity suggest that bioethicists should investigate how to distinguish such concepts. In some cases, the systematic review will simplify this task into that of deciding which of the entries in its list of very finely individuated reasons should be considered to be reasons of the same type.

Sixth, a systematic review of reasons may identify, as did ours, empirical assumptions that differ between publications, for example, assumptions about the cost, feasibility or legality of ensuring post-trial access to trial drugs. The empirical testing of these assumptions – which will require prior operationalization of appropriate concepts, e.g. of cost – will improve the information base of decisions, whether in practice or policy. For example, prominent international research ethics guidelines state that payments to research subjects should not be so large that they induce individuals to participate in research against their better judgment.^39^ Many ethics commentators argue that payments to research participants that do more than reimburse their costs should not be permitted because they may lead individuals to underestimate or undervalue the risks of participating.^40^ Limited evidence now shows that increasing payments to research participants is not associated with reducing the ability to assess risk.^41^ It may be that the case for the current guidelines on payments to research subjects remains compelling, even if the reason (apparently incorrectly) based on the effect of payments on risk assessment is removed from the case. Nonetheless, the legitimacy of a guideline based on a falsehood is questionable. A systematic review seems best placed to identify empirical assumptions that need testing.

Also, our systematic review aids bioethicists' identification of reasons relevant to determining whether there are other obligations, such as ensuring PTA to trial results,^42^ providing ART to participants who seroconvert in HIV vaccine trials,^43^ or providing participants with care not necessary to conduct the research, to prevent or address research-related injury, or to fulfil morally optional promises.^44^

For all the above reasons, a systematic review of reasons remains helpful even if some of the published reasons turn out to be irrelevant, or the implications of the reasons for the research questions have been misunderstood. We surmise that a systematic review of a young literature may hasten its maturation by identifying shortfalls and areas for further research. We surmise also that the main value of a systematic review of a mature literature is to identify the major trends and reversals in what will typically be a literature too vast, fragmented and complex for most decision-makers to collect and appraise. Possibly, the direct relevance to decision-makers of a systematic review of reasons increases as the field matures. As with any systematic review, decision-makers may lack the time to wait for one to be written. While ours was extremely time-consuming, the process should be speedier now that the methodology has been developed. That methodology, which we report elsewhere as a step-wise process,^45^ could be further automated, increasing its value to decision-makers. If the methodology is also applied to write reviews within large fields such as law or economics, the incentive to automate the process will increase and (amortized) cost decrease.^46^

The limitations of the literature revealed by our systematic review suggest that a systematic review that answers the question ‘should PTA be ensured to former participants in a clinical trial?’, as the only alternative model of SRs of reason-based literature proposes,^47^ would not be useful to decision-makers, because the answers given by many publications are unreliable. For the same reason, such a review would not place a burden of proof on those who disagree with the most common answer given in the literature to this question. Furthermore, all-things-considered conclusions may distract decision-makers, given that different weightings of values and different contexts could yield different conclusions. Because the aim of systematic reviews of reasons is not to present the literature's all-things-considered conclusion, they avoid giving decision-makers the impression that this conclusion is the correct one. Furthermore, a systematic review of conclusions that does not extract data on reasons will be uninteresting to philosophers, whatever the completeness or quality of the literature reviewed, as they will still need to ‘go back to first principles’: to consider all the relevant reasons, their implications for the specific issue and their relative weight.

A merely pragmatic consideration in favour of systematic reviews of reasons is that there is no need to measure the quality of reasoning: a complete answer does not require this. However, a key feature of the classical systematic review is ‘an assessment of the validity of the findings of the included studies’.^48^ If the relevant finding is that a specific reason has been published, the publication of the reason suffices to establish the validity of the finding. Thus, our model is less vulnerable to objections to the measurement of the quality of reasoning.

This consideration is superficial because, as noted, decision-makers will also need information on quality. Worse, one might object that decision-makers may confuse the most commonly-presented reasons with the strongest reasons, just as they may confuse the literature's all-things-considered conclusion (presented by a McCullough Model systematic review) with the truth. The explanation of why the most commonly-presented reasons may fail to be the strongest ones presumably varies with context, as mentioned above. Whatever the explanation, this objection threatens our view that, with regards to reason-based bioethics, systematic reviews of reasons are superior.

In reply, particularly because our systematic review showed that publications presenting the same common reason differed regarding its implications and persuasiveness, we consider it unlikely that readers would assume that the more commonly-presented reasons are the stronger ones. However, we concede that a common reason for a specific conclusion might be commonly presented, always endorsed, yet invalid. We therefore propose that systematic review methodology should be improved to enable it to identify possible conflicts of interest,^49^ and that – in the absence of a measure of quality – systematic reviews should warn readers against assuming that the more commonly presented reasons are the stronger reasons.^50^ Furthermore, research should also be conducted to understand whether or not such a warning suffices to prevent readers from making this assumption. The results should be used to assess whether the risk that invalid reasons will mislead policymakers is more, or less, serious than the risk that policymakers will fail to take into account potentially strong reasons that were excluded from the review because the literature presented them only as invalid reasons.

If it turns out that such a warning does not suffice, we recommend writing different systematic reviews for bioethicists versus policymakers. Bioethicists should be given all the published reasons, because this furthers their interest of identifying all the published reasons and because they are trained to assess reasons. Policymakers should, instead, be given a subset of the published reasons, that is, the strong reasons; if necessary, the data on how often the (strong) reasons were presented should be withheld. It may be necessary to construct a measure of quality; and this poses various challenges. For example, it will be necessary to consider whether the review should include only valid reasons, or the narrower set of reasons that are valid and sound. It will also be necessary to decide whether to include a reason when there is legitimate disagreement about its validity and soundness. It is important to note that, even in the absence of a measure of quality, the systematic reviews we advocate can be coupled with publications that discuss the quality and implications of the reasons identified by the systematic review.

### 4. Why *systematic* reviews?

As mentioned above, the rationale given by McCullough and colleagues for writing systematic reviews of reason-based bioethics is the same as in the case of classical systematic reviews, namely, that systematic reviews make possible maximally informed, minimally biased decisions. We add here that society has an interest in clinical decisions and policies that are maximally informed and minimally biased. Furthermore, various clinical decisions, policies and policy tools that appear to be based on solely factual considerations are in fact influenced by ethical considerations – in some cases ethical judgments that are implicit and unjustified.^51^

The rationale for writing systematic reviews of reason-based bioethics may, in some respects, be stronger than in the case of clinical epidemiology: reason-based bioethics is particularly contentious, and the reason-based bioethics literature is especially difficult to retrieve, not only for medical professionals, who are not trained to retrieve it, but also for reference librarians. This is because the literature is fragmented across different fields, a greater proportion of reason-based bioethics literature appears in books, and the literature has been inadequately indexed. A systematic review's complete reference list is an important aid for both decision-makers and scholars, obviating the need for hundreds of hours of hard-to-find, skilled labour to retrieve relevant publications. Furthermore, medical professionals may be better appraisers of clinical epidemiology than of reason-based bioethics. Also, a systematic review's summary of the positions taken by individual publications (reasons endorsed and any final conclusion reached) enables decision-makers to grasp key publications immediately without trawling through typically large amounts of text. Both these additional items – the complete reference list and summary of positions – save decision-makers time and greatly reduce the scope for error. Last, in the case of our particular systematic review at least, guidance is inconsistent and incomplete, and so an additional aid to decision-making is necessary. Admittedly, a systematic review makes more demands on decision-makers than an informal review, but its advantages justify these demands. Unlike the informal reviews and reports covered by our systematic review,^52^ our systematic review identifies all the empirical or ethical points of dissent and so sets the research agenda. Our systematic review's identification of stark differences between publications, for example, on matters of fact, shows that multi-disciplinary research is urgently needed to understand the costs,^53^ feasibility, legality and effects of requiring PTA, and any tendency of PTA offers to potential participants to boost recruitment or to reduce the quality of informed consent. Its identification of inter-publication differences in the formulation and implications of moral reasons suggests that further reason-based research is needed.

One might object that systematic reviews of reason-based bioethics are unnecessary to enable maximally informed, minimally biased decisions: informal reviews suffice. According to this view, it suffices to sample qualifying publications until no new reasons emerge from additional publications, a point that qualitative researchers call ‘theoretical saturation’. Arguably, this is standard philosophical practice, although philosophers neither use standard techniques to maximize the chance of retrieving all relevant material nor report reproducible searches.

We have already argued why a review of reasons must be based on an exhaustive search and explained the direct and indirect relevance to decision-making of a list of all the published reasons. We add here that appropriate aggregation of exhaustive data on reasons, and appropriate data on publications, can be combined to identify bias due to the fact that some arguments are published more frequently than others, in higher impact publications, or across a broader variety of journals. Our systematic review suggested that all these sources of bias were present. A systematic review of reasons has, due to its methodology, a much lower risk of bias than the individual publications it covers, which decision-makers would otherwise use to identify relevant reasons (or should use, in the absence of a systematic review of reasons). Bias minimization is crucial: most bioethics issues are highly contentious and stakeholders often have unequal power.

Furthermore, while much of the value of a systematic review of reasons lies in its list of all the published reasons, two other components are of direct use to decision-makers. One is its complete reference list, as mentioned previously. Particularly when the literature is mature and fragmented, decision-makers are likely to lack the time and skills to retrieve all the publications. The other is the table that summarizes the position taken by each included publication: the reasons it endorsed, and the all-things-considered conclusion reached. When a publication is long and difficult, such a table can greatly help decision-makers who wish to understand the positions taken by a specific publication. A systematic search seeks to be not only exhaustive but reproducible. Describing a reproducible search enables readers to assess whether the search is likely to have retrieved all relevant literature, and thus helps them reach the appropriate degree of confidence in the systematic review's findings. The analysis provided by a systematic review written according to our model should interest policymakers more than the limited analysis given in informal reviews. Only the former is designed to identify differences in the alleged implications of a reason or differences in how persuasive different publications find the reason. Furthermore, only the former provides rankings of reasons in terms of how frequently they were mentioned, endorsed, or rejected.

Thus, paradoxically, a systematic review of reasons is better placed than a systematic review of conclusions (as well as better placed than an informal review) to show both when we should take the field's all-things-considered conclusion seriously and when that conclusion places a burden of proof on those who hold different views.

### 5. Are reviews written according to our model really *systematic?*

The issue of whether our proposed reviews are properly called systematic is not merely definitional: systematic reviews have a special authority for clinical decision-makers and policymakers. It is therefore crucial to address possible objections.^54^

One objection is that although our proposed reviews do synthesize the literature, they do not draw the literature's all-things-considered conclusion to the question that the literature actually addresses. A systematic review, this objection continues, should include such a substantial synthesis, but our proposed reviews chiefly list the reasons that have been given for or against this view.

Our reply is that the reviews that we propose do indeed include a substantial synthesis: the list of reasons and their variants, and aggregate data for example on the number of narrow types of reason mentioned in the literature. Furthermore, our proposed synthesis better furthers the classical systematic review's aim of enabling maximally informed, minimally biased decision-making than a systematic review of conclusions. The latter may indeed jeopardize this aim by misleading decision-makers.

A second objection focuses on the lack of guidance our systematic reviews offer on the quality of reasons to policymakers facing ethical decisions. The objection points out that this makes them a considerably less useful tool than classical systematic reviews to policymakers facing decisions concerning, for example, which drug to use for a disease.

Our reply is that we agree that the systematic reviews we propose would be more useful if they assessed the quality of reasons. However, in the absence of evaluation, their methodology and the extent to which they have the potential to improve decision-making leads us to conclude that the second objection is not decisive. Furthermore, as mentioned, decision-makers can also be given careful analyses of individual reasons, for example, an analysis of reciprocity-based arguments for the view that former participants in ART trials should always have continued access to ART.^55^

A third objection arises when reviewing a body of literature, such as the literature on post-trial access, where the same term (for example reciprocity) is used by different publications to express different reason concepts (for example the concepts of reciprocity and of distributive justice). As we explain elsewhere,^56^ inconsistencies in the usage of language mean that one should not assign types of reason to reason occurrences on the basis of language alone: one must assign types partly on the basis of the concepts expressed by the language. Unfortunately, it sometimes happens that one author uses the word ‘reciprocity’ to express a concept that another author calls ‘distributive justice’, and some concepts such as reciprocity and distributive justice may actually overlap. Furthermore, there is reasonable disagreement about how to individuate concepts. Therefore, coding on the basis of concepts, instead of language, increases the chance that individual reviewers might code passages differently, as would different teams of reviewers. The risk of bias is thus substantially increased. The objection continues that a systematic review must have a minimal risk of bias, and so a review that codes reason occurrences on the basis of concepts cannot be systematic. If consistency of language usage is an indicator of the quality of literature, the problem arises that systematic reviews of the worst literatures, which presumably most need systematic reviews, are simply not feasible.^57^

The second objection does not apply when a literature's language usage is consistent, but we concede that there may be few such literatures. We would like to point out that, plausibly, a review's methodology can have substantial validity, although not optimal validity, when independent reviewers reach consensus on how to assign reason types or they appeal to an independent reviewer to resolve disagreement. In any case, we find it plausible to believe (in the absence of contrary evidence) that a multi-author systematic review will be more valid than the typical single-author informal review, which is written on the basis of an undocumented search and analysis. At least, the burden of proof falls on the single author of the informal review to disprove this. Furthermore, reviews written according to our model use most of the techniques of the classical systematic review. For example, they use pre-determined inclusion conditions and independent reviewers, as well as various quality control mechanisms. We should also remember that many classical systematic reviews have some bias.^58^

## CONCLUDING REMARKS

We have argued that systematic reviews of reason-based bioethics should not seek to answer an ethical question based on the quality-adjusted responses of the included publications. This is because such reviews may mislead decision-makers when a literature is incomplete, or when there are mutually incompatible, but individually reasonable answers to the ethical question. Furthermore, they can be written without identifying all the reasons given when the ethical questions are discussed, their alleged implications for the ethical question, and the attitudes taken to the reasons. However, we contended, there is a need for systematic reviews of reasons, which address the factual question of which reasons have been given when addressing an ethical question, and present detailed information on such reasons. We explained that systematic reviews of reasons potentially improve decision-making directly, in that their lists of published reasons, and of publications, best reduce the respective chances that there are relevant reasons, and publications, of which decision-makers are unaware. Also, their summary of positions taken – the reasons endorsed and any conclusion drawn by individual publications – enables decision-makers quickly and accurately to grasp publications, some of which are voluminous or unclear. Last, such reviews can improve decision-making indirectly, through the reliable identification of any necessary research that would improve the information-base and provision of research tools, and thus also improve the academic literature. However, we stressed, a systematic review of reasons cannot be the only item in a decision-makers' brief: this should also contain, among other items, a distillation of the best reasons that remains alive to the possibility of alternative, reasonable conclusions that can be drawn from individual reasons and the totality of reasons. Further research is needed on measuring the quality of reasons.

## Footnotes

1 Reason-based bioethics is sometimes called *philosophical* or *argument-based bioethics*. J. Ives &H. Draper.Appropriate Methodologies for Empirical Bioethics: It's All Relative.Bioethics2009; 23: 249–258; L.B. McCullough,J.H. Coverdale &F.A. Chervenak.Constructing a Systematic Review for Argument-based Clinical Ethics Literature: The Example of Concealed Medications.J Med Philos2007; 32: 65–76.

2 The reason-based bioethics literature has been written by philosophers, principlists, casuists, narrativists, and normatively-minded social scientists. We thank an anonymous reviewer for this point.

3 P. Borry,P. Schotsmans &K. Dierickx.Empirical Research in Bioethical Journals. A Quantitative Analysis.J Med Ethics2006; 32: 240–245.

4 N. Sofaer *et al*. Subjects' Views of Obligations to Ensure Post-trial Access to Drugs, Care, and Information: Qualitative Results from the Experiences of Participants in Clinical Trials (EPIC) Study.J Med Ethics2009; 35: 183–188.

5 G.H. Guyatt *et al*. GRADE: An Emerging Consensus on Rating Quality of Evidence and Strength of Recommendations.Br Med J2008; 336: 924–926.

6 J.P.T. Higgins & S. Green. eds. 2009. *Cochrane Handbook for Systematic Reviews of Interventions* Version 5.0.2 (updated Sep 2009). The Cochrane Collaboration. Available at: http://www.cochrane-handbook.org/[Accessed 3 Feb 2010].

7 L.A. Jensen &M.N. Allen.Meta-synthesis of Qualitative Findings.Qual Health Res1996; 6: 553–560; J. Popay,A. Rogers &G. Williams.Rationale and Standards for the Systematic Review of Qualitative Literature in Health Services Research.Qual Health Res1998; 8: 341–351; B. Lemmer,R. Grellier &J. Steven.Systematic Review of Nonrandom and Qualitative Research Literature: Exploring and Uncovering an Evidence Base for Health Visiting and Decision Making.Qual Health Res1999; 9: 315–328; E. Mills *et al*. (2005). Systematic review of qualitative studies exploring parental beliefs and attitudes toward childhood vaccination identifies common barriers to vaccination.J Clin Epidemiol; 58: 1081–1088; G.V. Glass &M.L. Smith.Meta-Analysis of Research on Class Size and Achievement.Ed. Eval. and Pol. Analysis.1979; 1: 2–16; R.J. Light & D.B. Pillemer. 1985. *Summing Up*. Cambridge, MA: Harvard University Press.

8 D. Strech,M. Synofzik &G. Marckmann.Systematic Reviews of Empirical Bioethics.J Med Ethics2008; 34: 472–477.

9 McCullough et al., *op. cit.* note 1; see also E.J. Emanuel et al. 2008. Introduction. In *The Oxford Textbook of Clinical Research Ethic.* E.J. Emanuel et al. New York: Oxford University Press: 3–6.

10 Strech et al., *op. cit.* note 8; D. Strech,M. Synofzik &G. Marckmann.How Physicians Allocate Scarce Resources at the Bedside: A Systematic Review of Qualitative Studies.J Med Philos2008; 33: 80–99; D. Strech *et al*. Are Physicians Willing to Ration Health Care? Conflicting Findings in a Systematic Review of Survey Research.Health Policy2009; 90: 113–124.

11 McCullough et al., *op. cit.* note 1.

12 McCullough et al., *op. cit.* note 1, p. 68.

13 Other possible outcomes presumably include the ethical permissibility or impermissibility of the intervention, and whether or not the intervention should be required by guidelines.

14 McCullough et al., *op. cit.* note 1, p. 69.

15 McCullough et al., *op. cit.* note 1, p. 67. We find the measure of adequacy confusing, because the summary score and score for the third feature measure the same thing: the quality of the ethical analysis and argument.

16 L.B. McCullough,J.H. Coverdale &F.A. Chervenak.Argument-based Medical Ethics: A Formal Tool for Critically Appraising the Normative Medical Ethics Literature.Am J Obstet Gynecol2004; 191: 1097–1102 (p. 1100). The paper expresses non-standard concepts of validity and soundness. A standard definition of validity holds that an argument is valid if and only if it is necessary that, if all the premises are true, then the conclusion is true (A.P. Martinich. 2005. *Philosophical Writing. An Introduction*. Malden: Blackwell Publishing: 20). Validity does not imply that the premises are true and does not imply non-circularity. However, for McCullough, a valid argument must be more than logically valid: it must also be the case that ‘relevant clinical and other facts [are] clearly identified and supported’ and that the argument comprises ‘a coherent whole’ (p. 1100). Similarly, ‘[a] sound argument is an argument which is valid and which contains only true premises’ (Martinich, *op. cit.* note 16, p. 20). But, for McCullough, a sound argument is, among other things, one with a conclusion on which the physician can act with confidence that patient care will be improved as a result. Such an argument must be more than sound: for example, it must also be relevant to the clinical decision.

17 The operational definition of *informal review* is given in our systematic review: N. Sofaer & D. Strech. Reasons why Post-trial Access to Trial Drugs Should, or Need not be Ensured to Research Participants: A Systematic Review. Unpublished Manuscript.

18 Decision-makers who may benefit from such systematic reviews include regulators, Institutional Review Boards (Research Ethics Committees) and designers of research protocols, for example researchers, sponsors of research, and Contract Research Organizations.

19 McCullough et al., *op. cit.* note 1, p. 73.

20 J.P.T. Higgins & D.G. Altman. eds. Chapter 8: Assessing risk of bias in included studies. In J.P.T. Higgins & S. Green. eds. *Cochrane Handbook for Systematic Reviews of Interventions* Version 5.0.1 (updated Sep 2008). The Cochrane Collaboration,2008. Available at http://www.cochrane-handbook.org[Accessed 3 Feb 2010]; J.P.T. Higgins & S. Green, eds. The Cochrane Collaboration. Available at: http://www.cochrane-handbook.org[Accessed 15 Feb 2010], section called ‘Key points’.

21 Sofaer & Strech, *op. cit.* note 17.

22 N. Sofaer & D. Strech, *op. cit.* note 17.

23 To be precise, in our systematic review, we recorded whether reason occurrences were used to argue that post-trial access should, or need not be provided.

24 D. Strech & N. Sofaer. How to Write a Systematic Review of Reasons. Unpublished manuscript; Sofaer & Strech, *op. cit.* note 17.

25 Strech & Sofaer, *op. cit.* note 24.

26 There is need for research to determine how best to distil and to construct a suitable measure of quality.

27 J.V. Lavery. 2008. The Obligation to Ensure Access to Beneficial Treatments for Research Participants at the Conclusion of Clinical Trials. In *The Oxford Textbook of Clinical Research Ethics*; E.J. Emanuel et al. New York: Oxford University Press: 697–708.

28 These wide disparities were only slightly reduced when types of reason were broadened to ensure that the disparity was not an artifact of having very narrowly individuated reasons. The informal reviews included in our systematic review were: R. Ashcroft. 2005. After the Trial is over: What are the Sponsor's Obligations? Available at: http://www.scidev.net/en/policy-briefs/after-the-trial-is-over-what-are-the-sponsor-s-obl.html[Accessed 6 July 2010]; A.L. Carse & M.O. Little. 2008. Exploitation and the Enterprise of Medical Research. In *Exploitation and Developing Countries: The Ethics of Clinical Research*. J.S. Hawkins & E.J. Emanuel, eds. Princeton & Oxford: Princeton University Press: 206–245; E. Chang.Fitting a Square Peg into a Round Hole? Imposing Informed Consent and Post-trial Obligations on United States Sponsored Clinical Trials in Developing Countries.South Calif Interdiscip Law J2002; 11: 339–360; E.J. Emanuel. 2008. Benefits to Host Countries. In *The Oxford Textbook of Clinical Research Ethics*. E.J. Emanuel et al., eds. Princeton & Oxford: Princeton University Press: 719–736; C. Grady.The Challenge of Assuring Continued Post-trial Access to Beneficial Treatment.Yale J Health Policy Law Ethics2005; 5: 425–435; B. Greenwood & W.P. Hausdorff. 2003. *After a Trial is Over: The Ethical Issues*. Available at: http://www.scidev.net/en/policy-briefs/after-a-trial-is-over-the-ethical-issues-1.html[Accessed 6 July 2010]; L.E. Hutt. Freebies for Subject 641: A Discussion of the Ethical Prospect of Providing Drug Trial Subjects with Post-trial Access to the Drug Tested – A Canadian Perspective. *Health Law J* 1998; 6 Spec No: 169–187; Lavery, *op. cit*. note 27; A.J. London. Responsiveness to Host Community Health Needs. In *The Oxford Textbook of Clinical Research Ethics*. E.J. Emanuel et al., eds. Princeton & Oxford: Princeton University Press: 737–744; R. Macklin. 2004. *Double Standards in Medical Research in Developing Countries*. Cambridge, UK: Cambridge University Press; J. Millum. Post-trial Access to Antiretrovirals: Who Owes What to Whom? *Bioethics* 2009. DOI: BIOT1736 [pii] 10.1111/j.1467-8519.2009.01736.x [doi]; National Bioethics Advisory Commission (NBAC), *op cit.* note 29; D. Orentlicher.Universality and its Limits: When Research Ethics can Reflect Local Circumstances.J Law Med Ethics2002; 30: 403–410; A.K. Page.Prior Agreements in International Clinical Trials: Ensuring the Benefits of Research to Developing Countries.Yale J Health Policy Law Ethics2002; 3: 35–66; Z. Zong.Should Post-trial Provision of Beneficial Experimental Interventions be Mandatory in Developing Countries?J Med Ethics2008; 34: 188–192.

29 Now disbanded. National Bioethics Advisory (NBAC) Commission. 2001. *Ethical and policy issues in international research: Clinical trials in developing countries*. Bethesda, MD: NBAC. Available at: http://bioethics.georgetown.edu/nbac/clinical/Vol1.pdf[Accessed 6 Jul 2010].

30 Nuffield Council on Bioethics (NCOB). 2002. *The ethics of research related to healthcare in developing countries.* London: NCOB. Available at: http://www.nuffieldbioethics.org/sites/default/files/Ethics%20of%20research%20related%20to%20healthcare%20in%20developing%20countries%20I.pdf[Accessed 6 Jul 2010].

31 We lack evidence that neglect of relevant reasons leads to poor decision-making. However, this seems to us to be a probable assumption, particularly when – as revealed by our systematic review of reasons –*many* and *entire classes* of published reasons are ignored. The content of the decision, which might be the selection of a policy, plausibly depends on the reasons considered. The fact that official bodies place such emphasis on collecting the relevant literature and giving voice to different groups of stakeholders suggest that they share this assumption. Furthermore, even if ignoring a relevant reason makes no difference to the *content* of the decision, it does make a difference to its perceived legitimacy. Particularly when the reason is associated with particular stakeholder groups, its omission will lay decision-makers open to the criticism that they ignored those stakeholders' perspective.

32 Of course, this is only true to the extent that such reasons are found by the systematic review. It is true that a systematic review increases the pool of findable reasons. However, since a systematic review relies on specific sources of data, such as particular databases, these in turn become the gate-keepers and must be scrutinized for comprehensiveness.

33 Admittedly, the risk arises that readers presented with what purports to be a comprehensive review will be less likely to wonder what it omits. The review's limitations must be clearly identified so that readers remain alive to the possibility that there may be relevant reasons which have never been published.

34 N. Daniels. 2008. *Just Health: Meeting Health Needs Fairly*. Cambridge, New York: Cambridge University Press.

35 It was also necessary to conduct an extensive consultation because the systematic review did not adequately capture the views, for example, of industry and patients, and because of the need for legitimacy. Further limitations are given in the text.

36 NBAC, *op. cit*. note 29; NCOB, *op. cit*. note 30; CIOMS. 2002. *International Ethical Guidelines for Biomedical Research Involving Human Subjects.* Geneva: Council for International Organizations of Medical Sciences. Available at: http://www.cioms.ch/publications/guidelines/guidelines_nov_2002_blurb.htm[Accessed 9 July 2010]; World Medical Association. 2008. World Medical Association Declaration of Helsinki: Ethical Principles for Medical Research involving Human Subjects. Ferney-Voltaire: World Medical Association. Available at: http://www.wma.net/en/30publications/10policies/b3/index.html[Accessed 9 July 2010].

37 Systematic reviews of reasons should be updated frequently, as is recommended in the case of classical systematic reviews. J.P.T. Higgins, S. Green & R.J.P.M. Scholten. 2008. Chapter 3: Maintaining Reviews: Updates, Amendments and Feedback. In *Cochrane Handbook for Systematic Reviews of Interventions* Version 5.0.1 (updated September 2008). J.P.T. Higgins & S. Green (eds). The Cochrane Collaboration. Available at: http://www.cochrane-handbook.org/[Accessed 3 Feb 2010].

38 CIOMS, *op. cit*. note 36; L.H. Glantz *et al*. Research in Developing Countries: Taking ‘Benefit’ Seriously.Hastings Cent Rep1998; 28: 38–42; Participants in the 2001 conference on ethical aspects of research in developing countries. Moral Standards for Research in Developing Countries: From ‘Reasonable Availability’ to ‘Fair Benefits’. *Hastings Cent Rep* 2004; 34: 17–27.

39 CIOMS, *op. cit.* note 36.

40 R. Macklin.On Paying Money to Research Subjects: ‘Due’ and ‘Undue’ Inducements.IRB1981; 3: 1–6; N. Dickert N &C. Grady.What's the Price of a Research Subject? Approaches to Payment for Research Participation.N Engl J Med1999; 341: 198–203.

41 S.D. Halpern.Towards Evidence Based Bioethics.BMJ2005; 331: 901–903.

42 C.V. Fernandez,E. Kodish &C. Weijer.Informing Study Participants of Research Results: An Ethical Imperative.IRB2003; 25: 12–19.

43 S. Berkley.Thorny Issues in the Ethics of AIDS Vaccine Trials.Lancet2003; 362: 992; C. Slack *et al*. Provision of HIV Treatment in HIV Preventive Vaccine Trials: A Developing Country Perspective.Soc Sci Med2005; 60: 1197–1208.

44 H.S. Richardson,L. Belsky.The Ancillary-care Responsibilities of Medical Researchers: An Ethical Framework for Thinking about the Clinical Care that Researchers Owe their Subjects.Hastings Cent Rep2004; 34: 25–33.

45 Strech & Sofaer, *op. cit.* note 24.

46 Automating a procedure to be applied more broadly will probably be more expensive than automating a more specific procedure. However, the former is likely to be less expensive to its end users, over whom the costs are amortized. We thank Reuben Thomas for this point.

47 McCullough et al., *op. cit.* note 1.

48 S. Green et al. 2008. Introduction. In *Cochrane Handbook for Systematic Reviews of Interventions* Version 5.0.1 (updated Sep 2008). J.P.T. Higgins & S. Green, eds. The Cochrane Collaboration. Available at http://www.cochrane-handbook.org[Accessed 15 Feb 2010], section 1.2.

49 A sufficiently large literature enables the testing of correlations between reasons presented (or endorsed) and author characteristics.

50 Even if a McCullough Model systematic review discloses that the literature's all-things-considered ethical conclusion may not be the truth, and the disclosure prevents readers from thinking that the conclusion is the truth, a systematic review of reasons is still preferable, in that only it presents the detailed data on reasons that decision-makers, scientists and academics need.

51 I. Miljeteig *et al*. Impact of Ethics and Economics on End-of-life Decisions in an Indian Neonatal Unit.Pediatrics2009; 124: 322–328; K.A. Johansson, B. Robberstad & O.F. Norheim. Further Benefits by Early Start of HIV Treatment in Low Income Countries: Survival Estimates of Early Versus Deferred Antiretroviral Therapy. AIDS Research and Therapy 2010; 7; D.W. Brock. 2003. Ethical Issues in the Use of Cost-effectiveness Analysis for the Prioritization of Health Care Resources. In *Making choices in health: WHO Guide to Cost-Effectiveness Analysis*. T. Tan-Torres Edejer et al. eds. Geneva: World Health Organization: 289–312; S. Anand &K. Hanson.Disability-adjusted Life Years: A Critical Review.J Health Econ1997; 16: 685–702; N. Sofaer *et al*. Is the Selection of Patients for Anti-retroviral Treatment in Uganda Fair? A Qualitative Study.Health Policy2009; 91: 33–42; D. Strech &J. Tilburt.Value Judgments in the Analysis and Synthesis of Evidence.J Clin Epidemiol2008; 61: 521–524; B. Molewijk *et al*. First the Facts, then the Values? Implicit Normativity in Evidence-based Decision Aids for Shared Decision-making.Z Evid Fortbild Qual Gesundhwes2008; 102: 415–420.

52 R. Ashcroft. After the Trial is over: What are the Sponsor's Obligations? 2005: Available at: http://www.scidev.net/en/policy-briefs/after-the-trial-is-over-what-are-the-sponsor-s-obl.html[Accessed 6 July 2010]; A.L. Carse & M.O. Little. 2008. Exploitation and the Enterprise of Medical Research. In *Exploitation and Developing Countries: The Ethics of Clinical Research*. J.S. Hawkins & E.J. Emanuel, eds. Princeton & Oxford: Princeton University Press: 206–245; E. Chang.Fitting a Square Peg into a Round Hole? Imposing Informed Consent and Post-trial Obligations on United States Sponsored Clinical Trials in Developing Countries.South Calif Interdiscip Law J 2002; 11: 339–360; E.J. Emanuel. 2008. Benefits to Host Countries. In *The Oxford Textbook of Clinical Research Ethics*. E.J. Emanuel et al., eds. Princeton & Oxford: Princeton University Press: 719–728; C. Grady.The Challenge of Assuring Continued Post-trial Access to Beneficial Treatment.Yale J Health Policy Law Ethics2005; 5: 425–435; B. Greenwood & W.P. Hausdorff. 2003. *After a Trial is Over: The Ethical Issues*. Available at: http://www.scidev.net/en/policy-briefs/after-a-trial-is-over-the-ethical-issues-1.html[Accessed 6 July 2010]; L.E. Hutt. Freebies for Subject 641: A Discussion of the Ethical Prospect of Providing Drug Trial Subjects with Post-trial Access to the Drug Tested – A Canadian Perspective. *Health Law J* 1998; 6 Spec No: 169–187; Lavery, *op. cit*. note 27; A.J. London. Responsiveness to Host Community Health Needs. In *The Oxford Textbook of Clinical Research Ethics*. E.J. Emanuel et al., eds. Princeton & Oxford: Princeton University Press: 737–744; R. Macklin. 2004. *Double Standards in Medical Research in Developing Countries*. Cambridge, UK: Cambridge University Press; J. Millum. Post-trial Access to Antiretrovirals: Who Owes What to Whom? *Bioethics* 2009. DOI: BIOT1736 [pii] 10.1111/j.1467-8519.2009.01736.x [doi]; National Bioethics Advisory Commission (NBAC), *op. cit.* note 29; D. Orentlicher.Universality and its Limits: When Research Ethics can Reflect Local Circumstances.J Law Med Ethics 2002; 30: 403–410; A.K. Page.Prior Agreements in International Clinical Trials: Ensuring the Benefits of Research to Developing Countries.Yale J Health Policy Law Ethics 2002; 3: 35–66; Z. Zong.Should Post-trial Provision of Beneficial Experimental Interventions be Mandatory in Developing Countries?J Med Ethics 2008; 34: 188–192.

53 Such multidisciplinary research should include the identification of an appropriate, measurable concept of cost as well as empirical research.

54 D. Strech. Evidence-based Ethics – What it Should Be and What it Shouldn't. *BMC Med Ethics* 2008; 9.

55 M. Merritt &C. Grady.Reciprocity and Post-trial Access for Participants in Antiretroviral Therapy Trials.AIDS2006; 20: 1791–1794.

56 Strech & Sofaer, *op. cit.* note 24.

57 We thank Leif Wenar for this point.

58 D. Moher *et al*. Preferred Reporting Items for Systematic Reviews and Meta-Analyses: The PRISMA Statement.Ann Intern Med2009; 151: 264–269.

